# Postoperative Administration of the Acetylcholinesterase Inhibitor, Donepezil, Interferes with Bone Healing and Implant Osseointegration in a Rat Model

**DOI:** 10.3390/biom10091318

**Published:** 2020-09-14

**Authors:** Faez Saleh Al-Hamed, Ola M. Maria, Jeff Phan, Ahmed Al Subaie, Qiman Gao, Alaa Mansour, Lina Abu Nada, Imane Boukhatem, Osama A. Elkashty, Simon D. Tran, Marie Lordkipanidzé, Zahi Badran, Faleh Tamimi

**Affiliations:** 1 Faculty of Dentistry, McGill University, Montreal, QC H3A0C7, Canada; Faez.al-hamed@mail.mcgill.ca (F.S.A.-H.); ola.maria@mail.mcgill.ca (O.M.M.); jeff.phan@mail.mcgill.ca (J.P.); ahmed.alsubaie@mail.mcgill.ca (A.A.S.); qiman.gao@mail.mcgill.ca (Q.G.); alaa.mansour@mail.mcgill.ca (A.M.); lina.abunada@mail.mcgill.ca (L.A.N.); osama.elkashty@mail.mcgill.ca (O.A.E.); simon.tran@mcgill.ca (S.D.T.); zahi.badran@mcgill.ca (Z.B.); 2 College of Dentistry, Imam Abdulrahman bin Faisal University, Dammam 34212, Saudi Arabia; 3 Faculté de Pharmacie, Université de Montréal, Montréal, QC H3T 1J4, Canada; imane-bkm@hotmail.fr (I.B.); marie.lordkipanidze@umontreal.ca (M.L.); 4 Research Center, Montreal Heart Institute, Montreal, QC H1T 1C8, Canada; 5 Faculty of Dentistry, Mansoura University, Mansoura 35516, Egypt; 6 Department of Periodontology (CHU/Rmes Inserm U1229/UIC11), Faculty of Dental Surgery, University of Nantes, 44042 Nantes, France; 7 College of Dental Medicine, University of Sharjah, Sharjah, P.O. Box 27272, UAE; 8 College of Dental Medicine, Qatar University, Doha P.O. 2713, Qatar

**Keywords:** acetylcholinesterase inhibitors, bone healing, osseointegration, donepezil, hemostasis

## Abstract

Donepezil is an acetylcholinesterase inhibitor commonly used to treat mild to moderate Alzheimer’s disease. Its use has been associated with increased bone mass in humans and animals. However, the effect of postoperative administration of donepezil on bone healing remains unknown. Therefore, this study aimed to assess the impact of postoperative injection of donepezil on bone healing, titanium-implant osseointegration, and soft tissue healing. Twenty-two Sprague-Dawley rats were randomly assigned to receive a daily dose of either donepezil (0.6 mg/kg) or saline as a control. In each rat, a uni-cortical defect was created in the right tibia metaphysis and a custom-made titanium implant was placed in the left tibiae. After two weeks, rats were euthanized, and their bones were analysed by Micro-CT and histology. The healing of bone defect and implant osseointegration in the rats treated with donepezil were significantly reduced compared to the saline-treated rats. Histomorphometric analysis showed lower immune cell infiltration in bone defects treated with donepezil compared to the saline-treated defects. On the other hand, the healing time of soft tissue wounds was significantly shorter in donepezil-treated rats compared to the controls. In conclusion, short-term administration of donepezil hinders bone healing whereas enhancing soft tissue healing.

## 1. Introduction

Bone remodeling is a continuous process of bone resorption by osteoclasts followed by bone formation by osteoblasts [1]. It is regulated locally through direct interactions between osteoclasts, osteoblasts, and immune cells, and centrally through three axes: the hypothalamic-pituitary-thyroid axis, the co-regulation of adipose tissue, bone tissue, and energy metabolism axis, and the IL-1 autonomic nervous system axis [2,3,4,5,6,7]. The latter two axes are mediated by both branches of the autonomic nervous system: the adrenergic and cholinergic branches. The adrenergic branch favors bone resorption, whereas the cholinergic branch favors bone formation [2].

The cholinergic system is regulated by the neurotransmitter, acetylcholine, an endogenous chemical that allows signal transmission between neurons [8]. Acetylcholine can activate nicotinic or muscarinic cholinergic receptors. The nicotinic receptors consist of α, β, γ, δ and ε subunits that form the ion channels. The muscarinic receptors are guanine nucleotide protein coupled receptors (m1, m2, m3, m4, and m5). Both muscarinic and nicotinic receptors are expressed on the membrane of bone cells and mesenchymal stem cells [9]. Among them, the nicotinic subtype-α2 receptor and muscarinic-3 (m3) receptor affect bone remolding [4,10]. Studies showed that knockout mice of nicotinic subtype-α2 receptors are osteoporotic due to osteoclast upregulation [4], whereas m3 knockout mice are osteoporotic due to reduced osteoblast numbers and increased osteoclast numbers [10]. The stimulation of muscarinic receptors in vitro showed an increase in osteoblast proliferation [11]. In addition, the cholinergic system increases bone mass directly by inducing osteoclast apoptosis through the activation of the nicotinic receptors and indirectly by inhibiting the sympathetic nervous system signaling [4].

The hypothalamic cholinergic receptors are degraded in elderly patients (e.g., Alzheimer’s disease (AD) patients) and thus may affect their bone health [12]. AD is the most common type of dementia affecting the elderly population and it is characterized by the neurodegeneration of the central nervous system (CNS). Acetylcholinesterase inhibitors (AChEIs) are a group of medications including Donepezil, Rivastigmine and Galantamine which are commonly used to treat mild to moderate cases of AD [13]. These drugs act as reversible acetylcholinesterase inhibitors that raise the concentration of acetylcholine at the neural synapses and result in improving patient’s memory [14]. In addition, these medications have been found to stimulate muscarinic cholinergic pathways and thereby have beneficial effects on bone metabolism [15].

A considerable number of elderly patients including Alzheimer’s patients are osteoporotic; they have reduced bone remodeling capacity and increased fracture risk [16]. Interestingly, the long-term use of AChEIs has been found to reduce the risk of hip fracture and enhance the outcomes of hip fracture surgeries in AD patients [17,18]. In addition, donepezil was found to reduce serum adrenaline level, a coagulation factor that affects hemostasis [15]. Hemostasis is required for bone and soft tissue regeneration and any disturbance in blood hemostasis may interfere with tissue healing. We hypothesized that cholinergic receptors can affect inflammation, coagulation, and bone formation, thus donepezil could affect bone healing by affecting hemostasis and inflammation. Therefore, we aimed to assess the effect of postoperative injection of donepezil on bone healing, soft tissue healing and hemostasis in a rat model. The use of acetylcholinesterase inhibitors by AD patients suffering from bone fractures has been associated with reduced risk of fracture non-union, improved bone quality, and fewer complications when compared to non-users [17]. Thus, understanding how postoperative administration of donepezil affects bone and soft tissue healing may have clinical implications in these patients.

## 2. Materials and Methods

This study was approved by McGill Animal Care and Use Committee (protocol # 2012–7269) in accordance with the Canadian Council for Animal Care guidelines.

### 2.1. Animals

A total of 30 healthy female (10–12 weeks-old) Sprague Dawley rats (Charles River Laboratories, Montreal, QC, Canada), weighing 200–250 g were purchased and housed (two animals per cage) in the Genome Animal Facility of McGill University. All animals were kept in a controlled environment at 22 °C and a humidity of 30–70% with 12-h light/dark cycles and were allowed to acclimatize to the new environment for two weeks prior to surgery. Water and a rodent diet were provided (ad libitum), and rats were monitored daily by a veterinarian in the animal facility. Out of the 30 rats, eight rats were used for hemostasis assessment and 22 rats underwent surgery to assess bone healing and implant osseointegration. Eight rats of the rats that underwent bone surgeries, were used for soft tissue healing assessment.

### 2.2. Bone Healing Assessment

**Figure 1:**
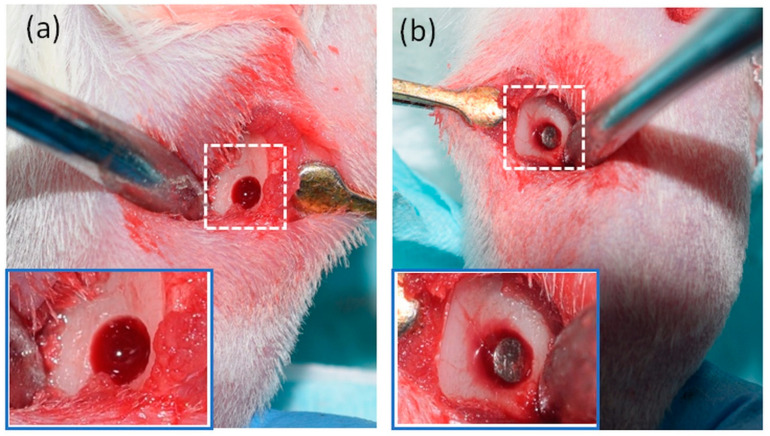
(**a**) A unicortical bone defect created in the lateral surface of the right tibial metaphysis. (**b**) A titanium implant placed in the lateral surface of the left tibial metaphysis.

Twenty-two rats were used for bone healing assessment. Bilateral tibial bone defects were conducted as previously described [19]. A unicortical defect (1.5 mm ø) was created in the left tibia, and a custom-made titanium implant (1.5 mm ø × 2.0 mm depth, McMaster-CARR; Aurora, OH, USA) was inserted. In the right tibia, a unicortical defect (2.5 mm ø) was created and was left empty. Postoperatively, the rats were divided randomly using a sealed envelope into two groups: the experimental group received daily subcutaneous injections of donepezil (0.6 mg/kg/day, Sigma-Aldrich) as described previously [15,20] and the control group received daily subcutaneous injections of saline (1 mL/kg/day). Rats were euthanized after two weeks using an overdose of CO_2_, and their tibiae were collected and analysed using micro-CT and histology (Figure 1).

Micro-computerized tomography (Micro-CT) analysis was conducted as described by Al Subaie et al., 2016 [19]. All tibiae with defects (n = 22) or titanium implants (n = 22) were scanned using a micro-CT (Sky-Scan1172; Bruker, Kontich, Belgium). Micro-CT image three-dimensional analysis was performed using the CTAn software (SkyScan 1172). The volume of interest of the bone samples was defined using a standardized algorithm as described in our previous studies [19,21,22]. Briefly, the volume of interest (VOI) was a cylinder that included the full diameter of the original defect (2.5 mm) and the thickness of the cortical bone surrounding the defect (Figure S1a,b). The cortical thickness was measured in coronal sections as shown in figure (Figure S1c). Bone architectures including the bone volume/tissue volume (BV/TV), the defect size, the trabecular thickness (Tb.Th), trabecular number (Tb.N), and trabecular separation (Tb.Sp)) were calculated within the VOI.

The peri-implant volume of interest (VOI) was determined as follows; first, the Ti implants were identified in 8-bit images by setting-up the greyscale index threshold ranging from 130 to 255. This threshold range was selected because it produced images that matched the real dimensions of the Ti implant as described in our previous study [19]. Second, using the “dilatation” tool of the CTAn software, the region within 50 µm of the implant perimeter was excluded from the analysis because it presented substantial noise caused by metal artefacts. Third, the peri-implant VOI selected for analysis was determined to be 50 to 70 µm away from the implant surface. This was done by subtracting a VOI, expanding 50 µm away from the Ti-implant, from a second VOI expanding 70 µm away from the Ti-implant. Within the selected peri-implant VOI, the bone was determined from 8-bit images in which the lower threshold was set at a greyscale index of 6 and the upper threshold at greyscale index of 255 (Figure S1d–g).These thresholds were selected because they were found to accurate identify mineralized bone in previous studies [21].

Histology and histomorphometry analyses of bone defects were conducted as previously described [19]. The right tibiae defects were dehydrated in ascending concentrations of ethanol (70–100%) using the automated paraffin tissue processor (ASP300-Leica, Wetzlar, Germany) and cleared with Xylene. Samples were pre-infiltrated with Paraplast X-Tra wax at 58 °C and embedded in paraffin wax (EG1160-Leica, Toronto, ON, Canada) and cut into 5 µm thick sections using a microtome (RM2265-Leica, Richmond Hill, Ontario, Canada). Three horizontal sections were obtained from each defect. Sections were stained with Hematoxylin and eosin (H&E) and Toluidine Blue (TB) stains to assess the number of chronic inflammatory cells (macrophages and lymphocytes) and mast cells respectively. Von Kossa stain was used to measure the percentage of new bone formation. The average number of mast cells per each square millimeter (mm^2^) were quantified using ZEN 2012 SP2 software (Zeiss, Jena, Germany) at a magnification of 40X. The chronic inflammatory cells were quantified using WEKA trainable segmentation plugin of the ImageJ software (Wayne Rasband; NIH, Bethesda, MD, USA) in which the region of interest in H&E stained sections were marked, followed by training the software in identifying the cells of interest by marking multiple immune cells. Thresholding adjustment was used to remove other types of cells, and finally a binary set-up was used to quantify the objects that represent the immune cells (Figure S2). Bone mineralization was assessed using ImageJ software by calculating the ratio of the new bone percentage divided by the total tissue area.

### 2.3. Soft Tissue Healing Assessment

Eight rats, underwent bone surgeries [saline recipient (n = 4) and donepezil recipient (n = 4)] as discussed above, were used for soft tissue assessment. In order to assess the effect of donepezil on wound healing, wounds in both legs were photographed on day 0, 2, 4, 8, 10, 12, and day 14. Wound healing was assessed using a modified scale based on wound color (red (necrotic), yellow, brown, or pink), surface regularity (regular or irregular), and wound edges (defined or ill-defined) [23]. The time required to heal was determined based on wound color and classified into two categories: healed (pink color) or non-healed (red, yellow, or brown color).

### 2.4. Platelet Function Testing

Eight rats that underwent bone surgeries as discussed above were used for platelet function assessment. At day 14 postoperatively, animals were sacrificed using intracardiac puncture, and their blood samples were collected in 3 mL Hirudin tubes (Mannheim, Germany). Whole blood platelet aggregation was assessed by impedance aggregometry (Multiplate^®^ Analyzer, Roche Diagnostics International Ltd.). Briefly, hirudinated blood was diluted (1:1) with NaCl 0.9% prior to addition of the platelet agonist. The following agonists were used: adenosine diphosphate (ADP, 20 µM), arachnoid acid (AA, 0.5 mM), collagen (5 µg/mL), and protease-activated receptor 4 activating peptide (PAR4, 500 µM). Platelet aggregation was measured at 37 ºC for 6 min and it was reported in arbitrary units (U) corresponding to the impedance area under the curve (AUC).

### 2.5. Assessment of Bleeding Time and Volume

The eight rats used for hemostasis assessment underwent sham surgeries; only the skin incision was done on both tibial bones of each rat. Afterwards, rats were randomly assigned to donepezil or saline treatments as discussed above. At day four postoperatively, bleeding time and volume were measured using the tail transection technique. Prior to tail transection, a ruler was used to mark 2 mm of the tail where the transection was performed using blade #11. After tail transection, its bleeding end was placed immediately into a plastic tube containing 2mL saline and monitored visually to determine when cessation of bleeding occurred. To determine bleeding volume, the total volume was measured after subtracting the original volume of saline. After bleeding cessation, rats were euthanized as mentioned previously.

### 2.6. Blinding

The retrieved bone samples, blood samples, and wound photos were labeled in a blinded manner. Micro-CT, soft tissue healing, platelet function, and bleeding time analyses were performed by a researcher blinded to the group allocation.

### 2.7. Statistical Analysis

The effect of donepezil on bone formation and implant osseointegration was set to be the primary outcome, whereas its effect on soft tissue healing and hemostasis was set as secondary outcomes. Sample size for the bone healing experiment was calculated to achieve a power of 80% at a significance level of 5% to be able to reject the null hypothesis that donepezil has no effect on bone healing and implant osseointegration. 10% difference between study groups was considered to be clinically relevant, and a 12% potential standard deviation was assumed based on our previous study [24]. Accordingly, a total of 10 rats per group were determined to be sufficient. However, one rat was added to each group to compensate for 10% potential losses. For the soft tissue healing assessment, a total of eight wounds per group were determined to be sufficient as described in a previous study [25]. All results were presented as mean ± SD. Normality of data was checked using Shapiro-Wilk test and all data were normally distributed. Student’s t test was used to compare study groups for all parameters. A *p*-value of < 0.05 was considered statistically significant.

## 3. Results

### 3.1. Donepezil Hinders Bone Healing

**Figure 2:**
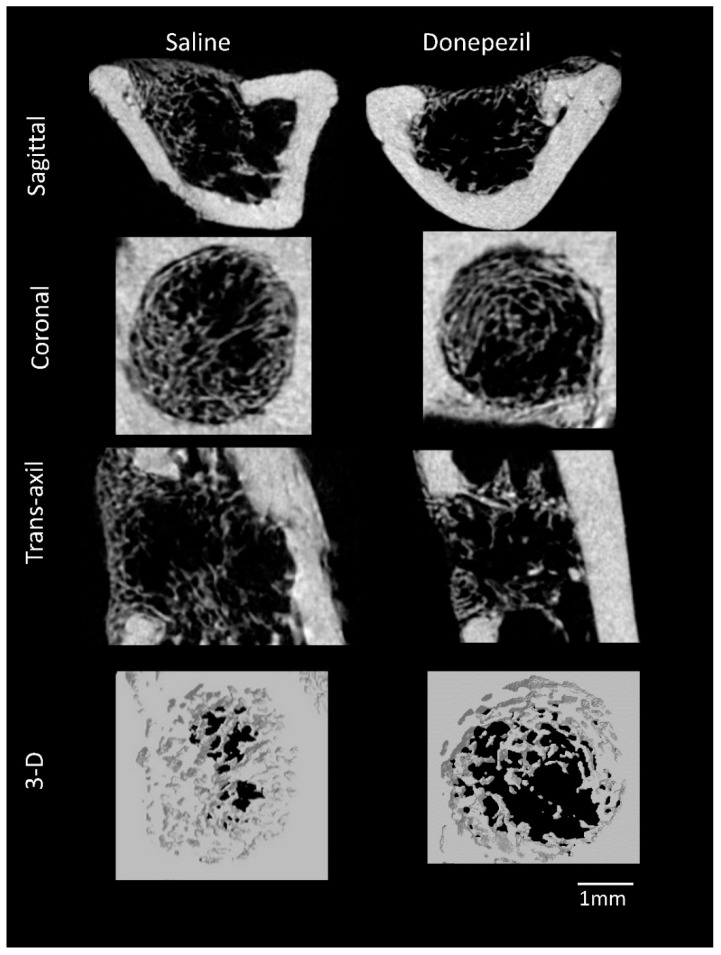
Sagittal, coronal, trans-axial, and 3-D µ-CT images of bone defects showing compromised bone healing in donepezil treated rats compared to saline treated rats.

**Figure 3:**
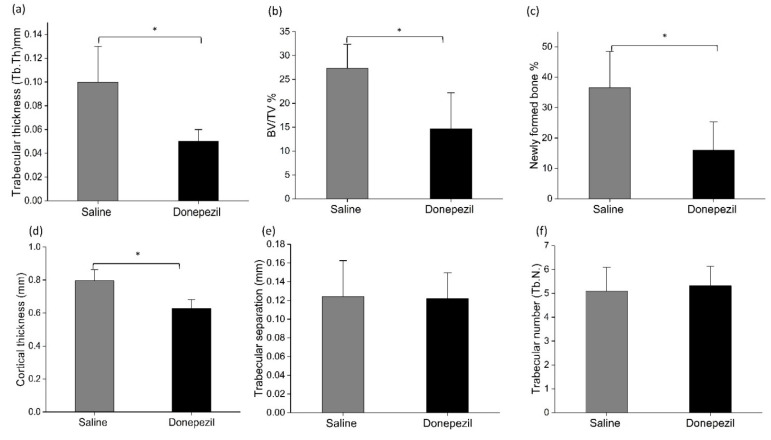
Micro-CT data analysis of bone defects in donepezil-treated rats compared to saline treated rats for the following parameters: trabecular thickness (Tb.Th), bone volume/tissue volume (BV/TV), percentage of new bone formation, cortical thickness, trabecular separation (Tb.Sp) and trabecular number (Tb.N). Statistical analysis was done using Student’s *t* test (n = 11 per each group). * indicates significant difference.

Micro-CT analysis of the bone defects showed that Donepezil-treated rats presented lower values in terms of bone volume/tissue volume (BV/TV) (14.7 ± 7.5% vs. 27.4 ± 5.0%; *p* = 0.003), trabecular thickness (0.05 ± 0.01 mm vs. 0.10 ± 0.03 mm; *p* = 0.001), cortical thickness (0.6 ± 0.05 mm vs. 0.8 ± 0.06 mm; *p* = 0.016), and the percentage of newly formed bone (16.0 ± 9.3% vs. 36.6 ± 11.8%; *p* = 0.002) compared to saline-treated rats. The trabecular number (Tb.N) and trabecular separation (Tb.Sp) were comparable in both groups [Tb.N: donepezil (5.3 ± 1.01/mm), saline (5.1 ± 1.01/mm), Tb.Sp: donepezil (0.13 ± 0.03/mm), saline (0.12 ± 0.03/mm); *p* ≥ 0.05] (Figure 2 and Figure 3).

**Figure 4:**
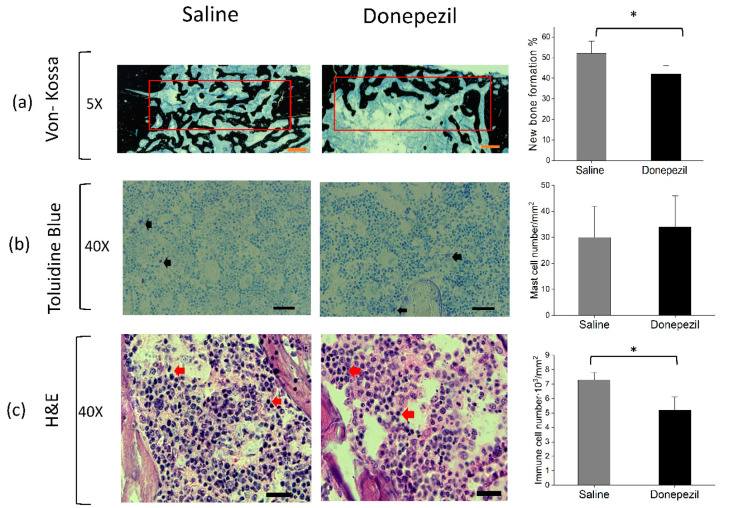
Histological analysis of bone defects. (**a**) Histological cross-sections stained with Von Kossa stain showing less mineralized newly formed bone in the donepezil group compared to the saline group (scale bar = 500 µm). Red rectangles represent the region of interest. (**b**) Histological cross sections of bone samples stained with Toluidine blue showing mast cell infiltration in donepezil-treated rats compared to saline-treated rats (scale bar = 20 µm). (**c**) Histological cross sections of bone samples stained with H & E showing chronic immune cells infiltration (lymphocytes & macrophages). Black and red arrows indicate the cells of interest. Bar charts represent the histomorphometric analyses. Data presented as mean ± SD. Statistical analysis was done using Student’s *t* test. * indicates significant difference.

Histomorphometric analysis showed that donepezil-treated defects presented significantly lower chronic immune cell infiltration (5.2∙10^3^ ± 0.9∙10^3^ vs. 7.3∙10^3^ ± 0.5∙10^3^ cell/mm^2^, *p* = 0.002, n = 14) and comparable mast cell infiltration (34 ± 12 vs. 30 ± 12 cell/mm^2^; *p* = 0.62, n = 14) compared to saline-treated defects. The donepezil group showed a lower percentage of new bone formation compared to the saline group (43 ± 6% vs. 52 ± 4%; *p* = 0.04, n = 12) (Figure 4).

### 3.2. Donepezil Interferences with Titanium-Implant Osseointegration

**Figure 5:**
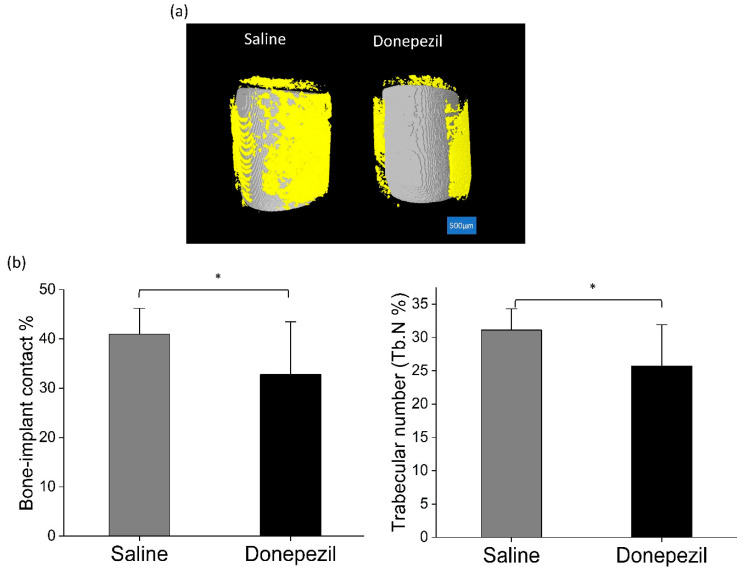
Micro-CT analysis of the implant. (**a**) 3-D μ-CT reconstructions of implants showing less bone (yellow) surrounding the implants (grey) in donepezil-treated rats compared to saline treated rats. Scale bar = 500 μm. (**b**) μ-CT data analysis of bone-implant contact % and trabecular number %. Statistical analysis was done using Student’s *t* test (n = 11 per each group). * indicates significant difference.

Micro-CT analysis of peri-implant area revealed that Donepezil-treated rats presented lower values of bone-implant BV/TV (32.8 ± 10.7% vs. 41.0 ± 5.2%; *p* = 0.03) and trabecular number (25.7 ± 6.2 vs. 31.1 ± 3.2% [1/mm]; *p* = 0.02) compared to saline-treated rats (Figure 5).

### 3.3. The Role of Donepezil on Soft Tissue Healing

**Figure 6:**
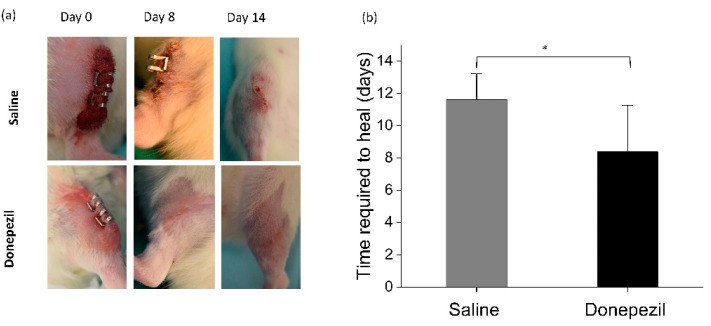
Time required to heal of skin wounds in donepezil and saline treated rats. (**a**) Photographs showing wound healing in donepezil and saline groups for day 0, 8, and day14 postoperatively. (**b**) Bar-chart showing faster healing time of donepezil treated wounds compared to controls. * indicates significant difference.

The average time required to heal was significantly shorter in donepezil-treated rats compared to the saline-treated ones (8.4 ± 2.9 days vs. 11.6 ± 1.7 days; *p* = 0.02) (Figure 6).

### 3.4. The Role of Donepezil on Hemostasis and Platelet Function

**Figure 7:**
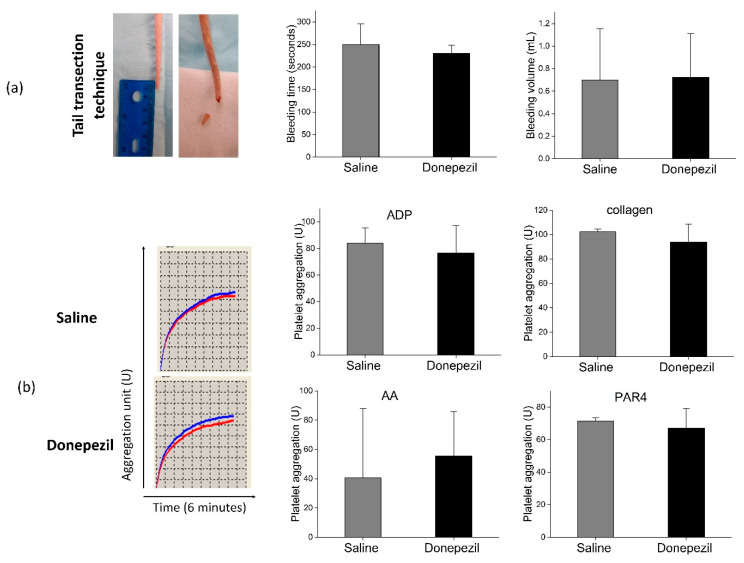
(**a**) Assessment of bleeding time and volume using tail transection technique. There was no significant difference between both groups (*p* > 0.05). (**b**) Platelet aggregation response to different agonists: adenosine diphosphate (ADP) (20 µM), arachnoid acid (AA) 500 µM, collagen, and protease-activated receptor 4 (PAR4), assessed by five channel multi-plate analyzer in donepezil and saline groups. Platelet aggregation response represents the area under the curve. X axis represents the test duration (6 min), Y axis represents the aggregation unit (U). Red and blue lines represent both electrodes and the platelet aggregation response to adenosine diphosphate (ADP, 20 µM) was shown as an example. There was no significant difference between both groups (*p* ≥ 0.05). Statistical analysis was done using Student’s *t* test (n = 4 rats per each group).

Bleeding time in donepezil-treated rats was 230 ± 19 s compared to 250 ± 46 s in saline-treated rats (*p* > 0.05). In addition, bleeding volume was comparable between both groups (0.7 ± 0.4 vs. 0.7 ± 0.5 mL). Platelet aggregation responses to ADP (77 ± 20 U vs. 84 ± 11U; *p* = 0.68), AA (56 ± 30 U vs. 41 ± 47 U; *p* = 0.0.68), collagen (94 ± 15 U vs. 103 ± 2 U; *p* = 0.49), and PAR4 activating peptide (67 ± 12 U vs. 72 ± 2 U; *p* = 0.66) in donepezil and saline-treated rats were comparable (Figure 7).

## 4. Discussion

An unexpected finding in this study was the fact that donepezil had opposing effects on skin healing and bone healing. These findings could be explained by differences between skin and bone healing in terms of the inflammatory processes, the cells involved, and the speed of the healing process.

During the early inflammatory phase of wound healing, immune cells and hematopoietic stem cells secrete pro-inflammatory cytokines such as interleukin-1 beta (IL-1 beta), interferon gamma (IFNγ), and tumor necrosis factor alpha (TNFα) [26,27,28]. TNFα and IFNγ have been found to enhance bone healing [29,30] and inhibit soft tissue healing [31,32]. Interestingly, donepezil is known to inhibit IFNγ and TNFα production [32,33,34]. This could partially explain why donepezil accelerated wound healing while it impaired bone healing.

Furthermore, donepezil may act differently on bone and skin cells [35,36]. Even though donepezil does not have a local direct effect on osteoprogenitor mesenchymal stem cell (MSCs) [37], acetylcholine could accelerate keratinocyte proliferation, migration, and viability [38]. This is mediated through the action of non-neuronal cholinergic receptors; muscarinic (M1 and M3) and a7 nicotinic receptors of keratinocytes [38,39]. Moreover, donepezil could further enhance skin wound healing through a feed-back loop, in which keratinocyte activation stimulates fibroblasts to release growth factors, which in turn stimulate keratinocyte proliferation [39,40].

Also, the healing time of bone defect lasts longer compared to skin wounds. Bone defects in rats take around 4-8 weeks to heal completely, and during this process, inflammation plays a predominant role during the first two weeks, while cell proliferation only kicks off in the second week. On the other hand, skin wounds have a short inflammatory phase and the proliferative phases kicks off as early as day three, when fibroblast starts to lay down the collagen matrix [41,42,43,44]. Given these differences in healing speed between skin and bone injuries, at the two-week time point of assessment in our study, bone healing would still have been under the influence of the inflammatory process, whereas skin healing would have been dominated by the proliferative stages of wound healing. This could also help explain why donepezil had opposite effects on skin healing and bone healing. Since donepezil is known to inhibit inflammation and stimulate cell proliferation, the two-week time point would have allowed us to perceive the positives effect on cell proliferation in skin healing but not in bone healing (Table S1).

The negative effect of donepezil on bone healing observed in our study could be seen to contradict the previous literature on the effect of donepezil on bone accrual. The use of donepezil has been associated with reduced risk of hip fracture and increased bone mass in animals, probably due to its effect on bone resorption during the remodeling process [15,16,17,18,45]. Furthermore, the use of AChEI was also associated with reduced risk of fracture complications in AD patients [45]. Thus, in the context of our results, we could hypothesize that the positive bone surgery outcomes associated with the long term use of AchEIs are probably due to better bone quality at the moment of the fracture compared to non-users [17], and this could outweigh the negative effects on bone healing. However, further studies investigating the effect of long-term donepezil administration on bone healing would be required to confirm this hypothesis.

Osseointegration and bone healing are similar processes that involve similar cells, cytokines and growth factors [46]. Therefore, drugs that reduce bone healing can also reduce osseointegration [47,48] and this is exactly what we observed herein. In our study, the bone-implant contact in the control group were comparable to previous studies [49,50]. However, donepezil decreased implant osseointegration compared to controls and this was similar to its effect on the healing of cortical bone defects. Those findings may have clinical implications in AD patients requiring orthopedic or maxillofacial surgeries particularly if they are requiring donepezil concurrently with the surgical procedure as donepezil interferes with the inflammatory phase of bone healing. Furthermore, bone implant contact is a well stablished indicator of mechanical stability of osseointegrated implants [51]. Thus, given its negative impact on osseointegration, donepezil would be expected to have also a detrimental effect on the mechanical stability of osseointegrated titanium implants. However additional mechanical studies would be required to confirm this.

Furthermore, Alzheimer’s disease patients are at high risk of developing osteoporosis. Reports suggest the use of prophylactic anti-osteoporosis therapies in AD patient [52]. These therapies include bisphosphonate and Denosumab, a human monoclonal antibody, which are commonly used for preventing or treating osteoporosis. They act by reducing bone turnover, improve bone mineral density, and consequently could reduce fracture risk [53,54]. However, their long-term use may associated with an increased fracture risk [55]. In terms of bone healing, in vivo studies showed that the use of antiosteoportic medications, including bisphosphonates, denosumab, calcitonin, estrogen, and raloxifene, do not impair endochondral fracture healing but they may delay repair due to impaired remodeling. However, bisphosphonates and denosumab delay callus remodeling, they increase callus volume and this results in improved biomechanical properties [56,57]. In the context of AD, the use of antiresorptive medications, taken together with or without AChEIs may have an impact on bone healing process. However, there is no in vivo studies assessing the effect of such treatment combination, thus further studies are required.

Acetylcholinesterase inhibitors (AchEIs) induce cholinergic effects on bone. Donepezil, rivastigmine, and galantamine increase bone mass indirectly via inhibiting the sympathetic activity on bone and directly via activating nicotinic and muscarinic receptors on bone cells. However, pyridostigmine was not associated with significant changes on bone mass [4]. Donepezil, rivastigmine, and pyridostigmine bind to both nAChERs and mAChERs, whereas galantamine binds to mAChERs only. High nicotine concentration is inhibitory for bone healing [58]. Therefore, increased concentrations of such drugs may desensitize nAChRs and interfere with bone healing.

Donepezil was found to rapidly increase brain acetylcholine signals which are mediated by muscarinic receptors, although these signals were desensitized despite the continuous increase of brain Ach concentration [59]. In addition, an in vivo study showed that injection of 1 mg/kg/day donepezil upregulates mRNA expressions of the cortex muscarinic receptors (M3, M4, M5) and a7 nicotinic receptors. In the current study, the donepezil dose was 0.6 mg/kg/day and it has been found to induce cholinergic effects by our previous study [15]. However, it is still unclear whether higher levels of acetylcholine within the cortex may or may not trigger the upregulation of cholinergic receptors [60]. This may indicate other mechanisms in addition to the central effect of donepezil, in which donepezil affect bone healing.

To further understand the mechanism by which donepezil hinders bone healing, we measured the effect of donepezil on hemostasis via measuring bleeding time, bleeding volume, and platelet function. We expected that donepezil may reduce platelet aggregation and consequently increase bleeding time and result in a weak platelet clot formed at the injury site. However, in our study, both donepezil and saline-treated rats showed comparable results. This indicates that donepezil does not affect initial hemostasis and it affects bone healing by different mechanism independent of hemostasis.

### Strengths, Limitations, and Future Directions

This study investigated the post-operative short-term effect of donepezil on bone using a well-established rat tibial bone defect model. This surgical model minimizes animal suffering while providing reliable and reproducible results regarding the assessment of the effect of medications on bone healing and implant osseointegration [61]. Nonetheless, future studies should be performed to further confirm our results in human patients and in more clinically relevant animal models such as bone fracture model or orthopedic osseointegrated devices.

One more limitation to be acknowledged was that the effect of donepezil on bone healing and osseointegration was assessed at a single time point, two weeks after surgery. At this time point, the control rats presented around 37% reduction in the volume of original defect. Thus, given the speed of bone healing in young rats, it is possible that at longer time points (i.e., four weeks), bone defects among controls would have completely heal, and thus hindering potential comparisons between groups. In addition, this period allows an accurate assessment of different medications on the inflammatory and proliferative phases of bone healing in terms of bone cells quantity and function [61]. However, further studies are required to study the long-term effect of donepezil on bone healing and implant osseointegration.

## 5. Conclusions

Short term postoperative administration of donepezil reduced bone defect healing and implant osseointegration in rats’ tibiae, whereas it improved soft tissue healing. Donepezil administration did not affect hemostasis and platelet function.

## Supplementary Materials

The following are available online at https://www.mdpi.com/2218-273X/10/9/1318/s1, Figure S1: The methodology used for bone defect and implant analyses, Figure S2: Validation of WEKA plugin in identifying chronic immune cell infiltration using ImageJ software, Table S1: The main differences between bone and skin wound healing.
