# Supplementary material for: Postoperative Administration of the Acetylcholinesterase Inhibitor, Donepezil, Interferes with Bone Healing and Implant Osseointegration in a Rat Model
Source: Biomolecules. 2020 Sep 14;10(9):1318. doi: 10.3390/biom10091318 (PMC7563209; doi:10.3390/biom10091318)
Supplement: Supplementary file 1 [file biomolecules-10-01318-s001.pdf]

Supplementary materials

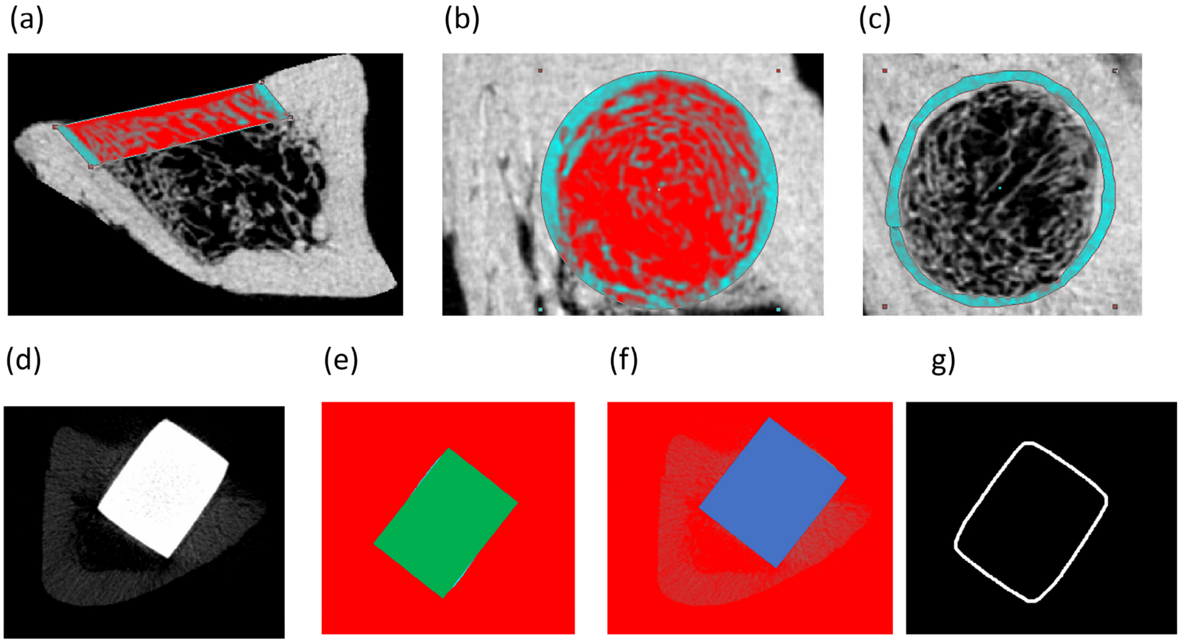

Figure S1. The methodology used for bone defect and implant analyses. (**a,b**) Sagittal and coronal images of the bone defect, showing the selected region of interest (red) used for the analysis of the bone defect. (**c**) A coronal image of bone defect, showing the region of interest (blue) used to measure the cortical thickness. (**d**) A sagittal image of the implant and its surrounding bone. (**e**) Ti implant was defined as the first volume of interest (VOI). (**f**) The Ti implant and the surrounding bone were identified as the second VOI. (**g**) The first VOI was subtracted from the second VOI and the surrounding bone was analyzed. The analysis was performed using CT-Analyser software (SkyScan, 1172).

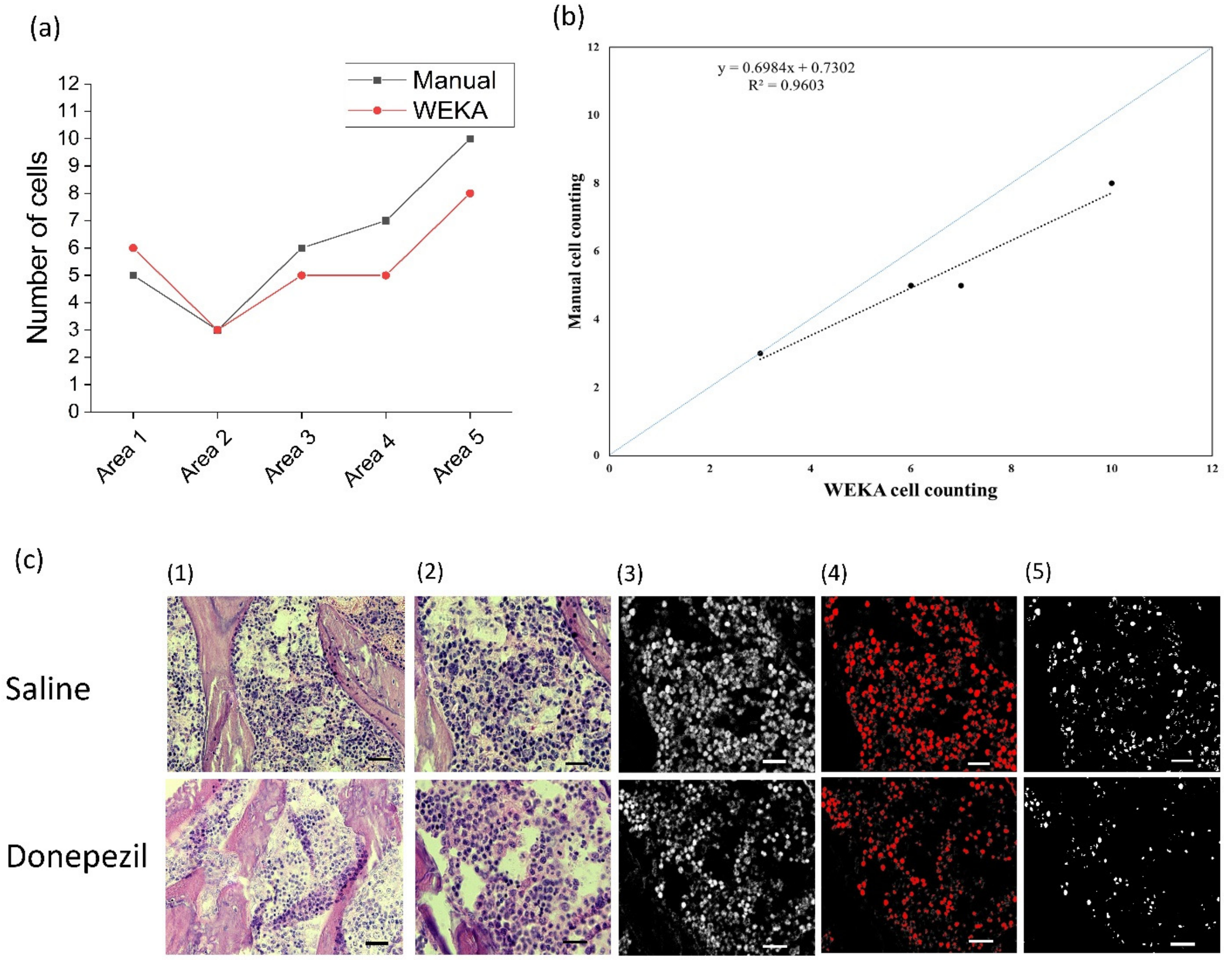

Figure S2. Validation of WEKA plugin in identifying chronic immune cell infiltration using ImageJ software. (**a**) Manual and trainable cell counting (WEKA) were performed in 5 regions of interest. (**b**) Linear relationship between WEKA and manual cell counting methods. The dotted black line represents the linear fitting of the data (R2 = 0.96). (**c**) Steps for analysis of chronic immune cells (lymphocytes & macrophages) infiltration using WEKA trainable segmentation plugin of ImageJ software. (1) H&E stained sections were opened using Image J software. (2) A photograph showing the cropped region of interest. (3) Determination of the probability maps. (4) Thresholding adjustment were used to remove other types of cells. (5) Binary set up (the program quantifies only the white objects (Magnification = 40X, Scale bar = 20 μm).

Table S1. The main differences between bone and skin wound healing.

**Parameter**	**Bone healing**	**Soft tissue healing**	**Ref**
**Phases of healing**	Proliferation phase involves chondrogenesis, osteogenesis, and angiogenesis	Proliferation phase involves fibroplasia, reepithelialization, and angiogenesis.	[1–5]
	Proliferation phase start at day 7-10 after injury.	Proliferation phase start at day 3 after injury.	
**Main Cells involved**	Osteoblasts, osteoclasts, osteocytes, endothelial cells, platelets, and immune cells	Fibroblasts, keratinocytes, epithelial cells, melanocytes, platelets, and immune cells	[1,6]
**Main sources of progenitor cells**	Periosteum, bone marrow, and haematopoietic progenitor cells	Bone marrow, haematopoietic progenitor cells, and connective tissue sheath of murine hair follicle	[7,8]
**Extracellular matrix**	Secreted mainly by osteoblasts	Secreted mainly by fibroblasts	[3,9]
**Healing time**	Longer healing time (4-8 weeks in rats)	Shorter healing time (1-3 weeks in rats)	[16–18].
**Scar tissue formation**	No	Yes (remodeled with time)	[19,20]

References:

1. Marsell, R.; Einhorn, T. The biology of fracture healing. *Inj.* **2011**, *42*, 551–555, doi:10.1016/j.injury.2011.03.031.
2. Cañedo-Dorantes, L.; Cañedo-Ayala, M. Skin Acute Wound Healing: A Comprehensive Review. *Int. J. Inflamm.* **2019**, *2019*, 3706315–15, doi:10.1155/2019/3706315.
3. Le, A.D.; Brown, J.J. Chapter 2 - Wound Healing: Repair Biology and Wound and Scar Treatment, in: Bagheri, S.C.; Bell, R.B.; Khan, H.A. (Eds.), Current Therapy In Oral and Maxillofacial Surgery, W.B. Saunders, Saint Louis, 2012, pp. 6-10.
4. Landén, N.X.; Li, D.; Ståhle, M. Transition from inflammation to proliferation: a critical step during wound healing. *Cell. Mol. Life Sci.* **2016**, *73*, 3861–3885, doi:10.1007/s00018-016-2268-0.
5. Marsell, R.; Einhorn, T. The biology of fracture healing. *Inj.* **2011**, *42*, 551–555, doi:10.1016/j.injury.2011.03.031.
6. Sorg, H.; Hager, S.; Hauser, J.; Mirastschijski, U.; Tilkorn, D.J. Skin Wound Healing: An Update on the Current Knowledge and Concepts. *Eur. Surg. Res.* **2016**, *58*, 81–94, doi:10.1159/000454919.
7. Ono, N.; Kronenberg, H.M. Bone repair and stem cells. *Curr. Opin. Genet. Dev.* **2016**, *40*, 103–107, doi:10.1016/j.gde.2016.06.012.
8. Lau, K.; Paus, R.; Tiede, S.; Day, P.; Bayat, A. Exploring the role of stem cells in cutaneous wound healing. *Exp. Dermatol.* **2009**, *18*, 921–933, doi:10.1111/j.1600-0625.2009.00942.x.
9. Curry, A.S.; Pensa, N.W.; Barlow, A.M.; Bellis, S.L. Taking cues from the extracellular matrix to design bone-mimetic regenerative scaffolds, *Matrix Biol.: journal of the International Society for Matrix Biology* **2016**, *52-54*, 397-412, doi:10.1016/j.matbio.2016.02.011.
10. Glass, G.E.; Chan, J.K.; Freidin, A.; Feldmann, M.; Horwood, N.J.; Nanchahal, J. TNF- promotes fracture repair by augmenting the recruitment and differentiation of muscle-derived stromal cells. *Proc. Natl. Acad. Sci.* **2011**, *108*, 1585–1590, doi:10.1073/pnas.1018501108.

11. Takayanagi, H.; Ogasawara, K.; Hida, S.; Chiba, T.; Murata, S.; Sato, K.; Takaoka, A.; Yokochi, T.; Oda, H.; Tanaka, K.; et al. T-cell-mediated regulation of osteoclastogenesis by signalling cross-talk between RANKL and IFN-γ. *Nat.* **2000**, *408*, 600–605, doi:10.1038/35046102.
12. Ashcroft, G.S.; Jeong, M.-J.; Ashworth, J.J.; Hardman, M.J.; Jin, W.; Moutsopoulos, N.; Wild, T.; McCartney-Francis, N.; Sim, D.; McGrady, G.; et al. Tumor necrosis factor-alpha (TNF-α) is a therapeutic target for impaired cutaneous wound healing. *Wound Repair Regen.* **2011**, *20*, 38–49, doi:10.1111/j.1524-475X.2011.00748.x.
13. Laato, M.; Heino, J.; Gerdin, B.; Kahari, V.M.; Niinikoski, J. Interferon-gamma-induced inhibition of wound healing in vivo and in vitro, *Ann. Chir. Gynaecol.* 90 Suppl **2001**, *215*, 19-23.
14. Hoff, P.; Gaber, T.; Strehl, C.; Schmidt-Bleek, K.; Lang, A.; Huscher, D.; Burmester, G.R.; Schmidmaier, G.; Perka, C.; Duda, G.N.; et al. Immunological characterization of the early human fracture hematoma. *Immunol. Res.* **2016**, *64*, 1195–1206, doi:10.1007/s12026-016-8868-9.
15. Mirza, R.E.; Fang, M.M.; Ennis, W.J.; Koh, T.J. Blocking Interleukin-1β Induces a Healing-Associated Wound Macrophage Phenotype and Improves Healing in Type 2 Diabetes. *Diabetes* **2013**, *62*, 2579–2587, doi:10.2337/db12-1450.
16. Lim, J.; Lee, J.; Yun, H.-S.; Shin, H.-I.; Park, E.K. Comparison of bone regeneration rate in flat and long bone defects: Calvarial and tibial bone. *Tissue Eng. Regen. Med.* **2013**, *10*, 336–340, doi:10.1007/s13770-013-1094-9.
17. Song, S.-H.; Yun, Y.-P.; Kim, H.J.; Park, K.; Kim, H.J.; Song, H.-R. Bone Formation in a Rat Tibial Defect Model Using Carboxymethyl Cellulose/BioC/Bone Morphogenic Protein-2 Hybrid Materials. *BioMed Res. Int.* **2014**, *2014*, 1–8, doi:10.1155/2014/230152.
18. Martins, N.L.P.; Malafaia, O.; Ribas-Filho, J.M.; Heibel, M.; Baldez, R.N.; De Vasconcelos, P.R.L.; Moreira, H.; Mazza, M.; Nassif, P.A.N.; Wallbach, T.Z. [Healing process in cutaneous surgical wounds in rats under the influence of Orbignya phalerata aqueous extract]. *Acta Cir. Bras.* 2006, *21*, 66–75.
19. Schell, H.; Duda, G.N.; Peters, A.; Tsitsilonis, S.; Johnson, K.A.; Schmidt-Bleek, K. The haematoma and its role in bone healing. *J. Exp. Orthop.* **2017**, *4*, 5, doi:10.1186/s40634-017-0079-3.
20. Xue, M.; Jackson, C.J. Extracellular Matrix Reorganization During Wound Healing and Its Impact on Abnormal Scarring. *Adv. Wound Care* **2015**, *4*, 119–136, doi:10.1089/wound.2013.0485.
